# Malignant Proliferating Trichilemmal Tumour Presenting Early in Life: An Uncommon Feature

**DOI:** 10.4103/0974-2077.79196

**Published:** 2011

**Authors:** Shalinee Rao, Ramya Ramakrishnan, D Kamakshi, Sibi Chakravarthi, Sandhya Sundaram, Duvuru Prathiba

**Affiliations:** *Department of Pathology, Sri Ramachandra University, Porur, Chennai, India*; 1 *Department of Surgery, Sri Ramachandra University, Porur, Chennai, India*; 2 *Department of Plastic Surgery, Sri Ramachandra University, Porur, Chennai, India*

**Keywords:** Malignant proliferating trichilemmal tumour, scalp, young

## Abstract

Malignant proliferating trichilemmal tumour is a rare cutaneous malignant neoplasm usually occurring in elderly women. We present a case of malignant trichilemmal tumour in a young lady of 26 years of age with a previous history of proliferating trichilemmal tumour at the same site.

## INTRODUCTION

Malignant proliferating trichilemmal tumour (MPTT) is a rare cutaneous tumour predominantly affecting the scalp, eyelids, neck and face. A review of the literature reveals MPTT to be a neoplasm of the older age group.[1,2] We present a case of MPTT occurring in a young lady.

## CASE REPORT

A 26-year-old lady presented with a history of swelling behind her right ear of seven months’ duration. There was no history of any discharge or constitutional symptoms.

She gave a history of a similar swelling in the same region (right post-auricular) for which she underwent excision and split-skin grafting nine months ago. On histopathology, it was found to be a proliferating trichilemmal tumour. The lesion recurred within two months of excision and increased to the present size in seven months. There was a rapid increase in size in the last two months. The patient also gave a history of another swelling in the right parieto-occipital region which was excised two years ago and was diagnosed as a benign trichilemmal tumour.

**Figure 1:**
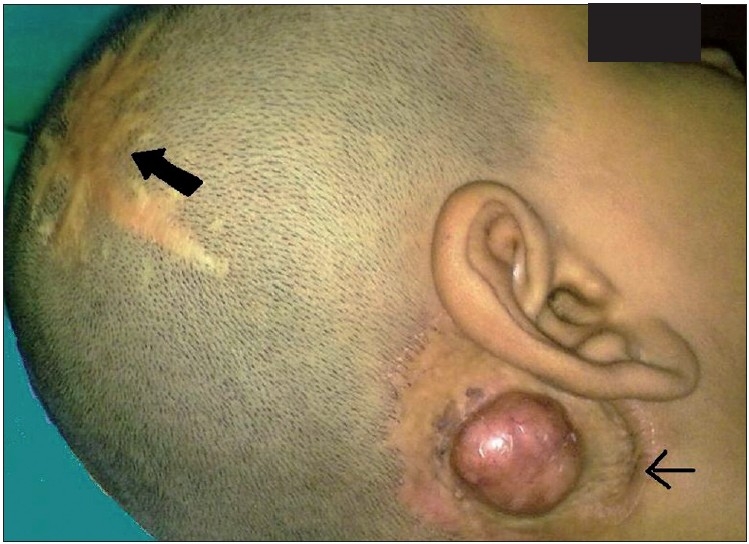
Right posterior auricular swelling with a healed curvilinear scar (thin arrow) over the scalp; Note another irregular healed scar at the right parieto-occipital region (thick arrow)

**Figure 2:**
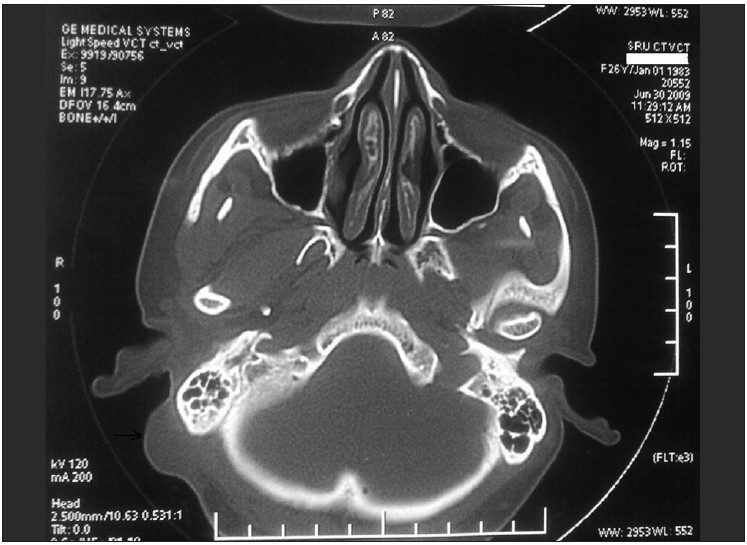
CT scan shows soft tissue mass with uninvolved mastoid bone

On examination, there was a 2.5 × 2 cm hemispherical swelling in the right post-auricular region, with an overlying scar of the previous split-skin grafting [Figure 1]. It was non-tender, firm in consistency and was not mobile. The skin was not pinchable. A differential diagnosis of recurrent trichilemmal tumour, keratoacanthoma and cylindroma was considered. A linear well-healed scar was also noted in the right parieto-occipital region [Figure 1]. There was no regional lymphadenopathy. To evaluate the underlying bone involvement and the exact extent of tumour, radiological workup was done with X-ray skull and computed tomography (CT) scan which showed no bone involvement [Figure 2].

### Surgery details

**Figure 3:**
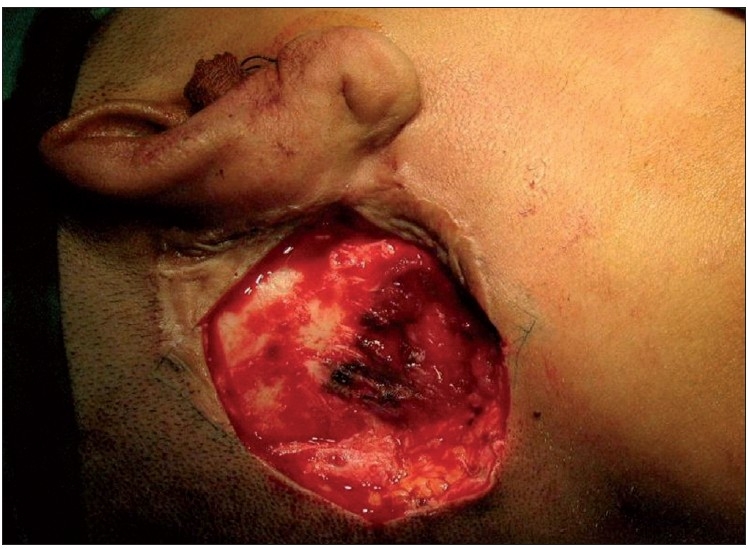
Mastoid bone shaved for a better surgical clearance

Under general anaesthesia with endotracheal intubation, patient in the supine position with the head turned towards the left, a rhomboid-shaped incision was made around the tumour. Wide excision was done giving a 2 cm clearance all around. The underlying mastoid was shaved as a precautionary measure [Figure 3]. A transposition flap from the neck was used to cover the defect in the right post-auricular region. The postoperative course was uneventful and the patient is doing well on follow-up.

### Histopathological examination findings

Gross examination of the excised skin showed a centrally positioned nodule measuring 2.2 cm in diameter just beneath the skin. Grossly, the overlying skin, circumferential and deep resected margins were not involved by the lesion.

**Figure 4:**
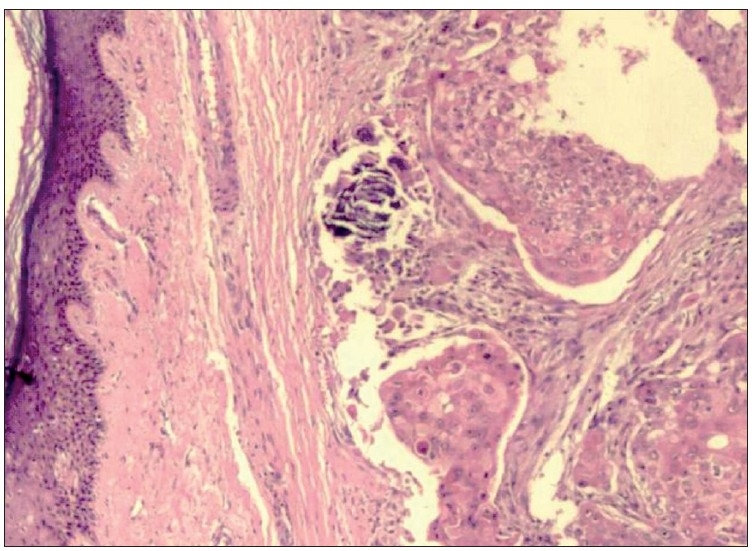
Skin with tumour islands in the dermis arranged in lobular and diffuse pattern (H and E, 20×)

**Figure 5:**
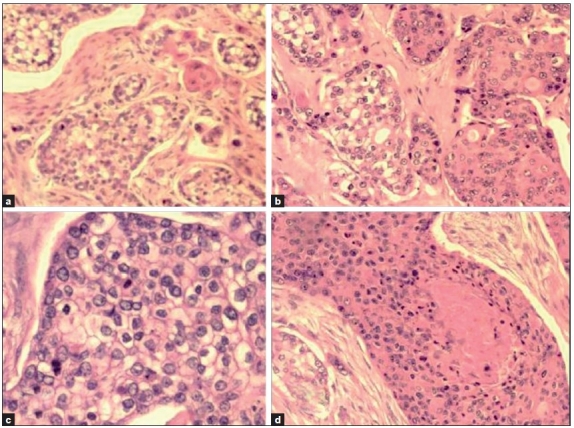
a- Tumour cells arranged in lobules with a few showing keratinization (H and E, 40×); b- Individual tumour cells are polygonal with some showing clearing of cytoplasm and keratinization (H and E × 40); c: Islands of clear tumour cells showing nuclear pleomorphism and mitotic activity (H and E, 100×); d: Neoplastic cells show abrupt keratin production (H and E, 40×)

**Figure 6:**
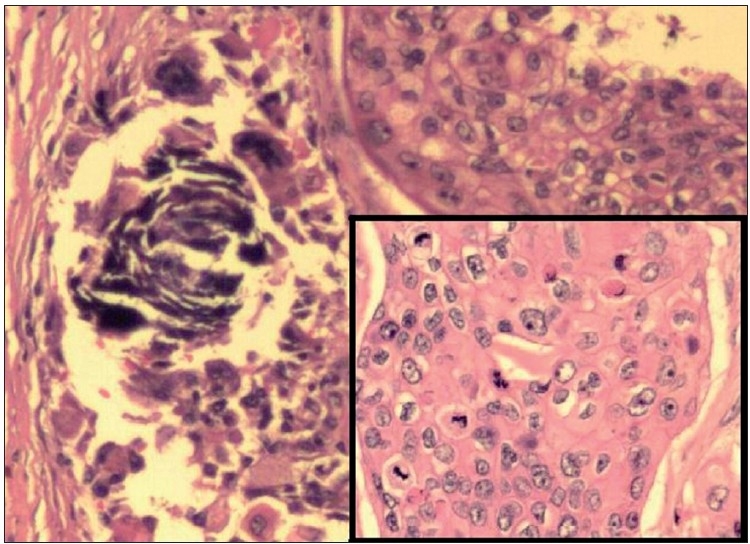
Tumour nests showing basophilic areas of calcification (H and E, 100×); Inset shows islands of pleomorphic tumour cells with many mitotic figures, a few of which appear atypical (H and E, 200×)

Histopathological examination of haematoxylin and eosin (H and E)-stained sections from the skin showed a tumour located in the dermis. The tumour cells were arranged as islands, in lobular and diffuse pattern [Figures 4 and 5a]. Neoplastic cells were oval to polygonal with abundant cytoplasm and many showed individual cell keratinization [Figure 5b]. The predominant population of tumour cells showed clearing of cytoplasm with high nucleo-cytoplasmic ratio, nuclear hyperchromasia and moderate nuclear pleomorphism [Figure 5b and c]. Tumour lobules showed abrupt keratinization [Figure 5d] and calcification [Figure 6]. Some of the tumour cells appeared bizarre. Numerous mitotic figures were also noted [Figure 6 inset]. Occasional focus showed foreign body giant cell reaction. On microscopic examination, skin, soft tissue and bony margins were free of tumour.

**Figure 7:**
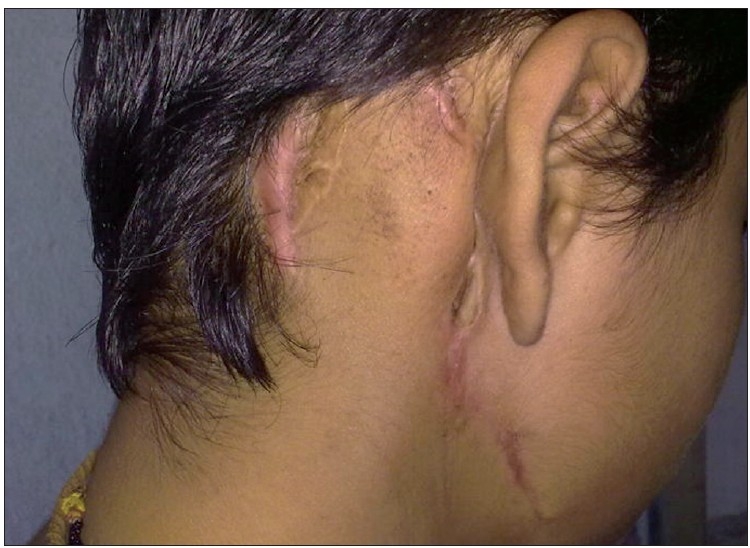
One year postoperative image showing healed scar

Final diagnosis on histopathological examination was malignant proliferating trichilemmal tumour. One year after the surgery, patient is doing well with no recurrence [Figure 7].

## DISCUSSION

Malignant proliferating trichilemmal tumour is an extremely uncommon tumour with differentiation towards hair and/or follicle.[3] The term MPTT was originally described in 1983 by Saida *et al*., and its biological behaviour still remains unpredictable.[4] There are conflicting statements with regards to description of proliferating trichilemmal tumour (PTT) and MPTT, their treatment and behaviour.[5] In a study done on a large series of pilar tumours, categorization was attempted based on histomorphology. Group I consisted of benign cases showing tumour cells with minimal nuclear atypia, trichilemmal keratinization, mononuclear inflammatory infiltrate and dystrophic calcification in the stroma. This group showed no tumour cell infiltration into the surrounding stroma. Group II with early invasive property showed moderate cytological atypia and stromal oedema with a mild to moderate infiltration of mononuclear inflammatory cells and were considered as locally aggressive. Group III tumours were frankly invasive with cellular anaplasia and were considered malignant.[6]

Proliferating trichilemmal tumour is a benign neoplasm that may be multiple as noticed in our case and it can rarely undergo malignant transformation in a step-wise manner starting with an adenomatous stage of the trichilemmal cyst to an epitheliomatous stage of the PTT evolving into carcinomatous stage of the MPTT.[4] This tumour lacks a distinctive histological or immunohistochemical marker to suggest malignant transformation. Increased proliferation index and DNA aneuploidy in tumour cells is an expression of a premalignant event.[7] Clinically sudden enlargement of longstanding nodular scalp lesions and histological evidence of significant abnormal mitosis, marked cellular pleomorphism, infiltrating margins and aneuploidy reflect malignant transformation. Our case possibly represents the event of malignant transformation in a proliferating trichilemmal tumour. Demonstration of regional or distant metastasis is considered an unequivocal sign of malignancy.[8]

It may be difficult to distinguish MPTT from squamous cell carcinoma and trichilemmal carcinoma (TLC) which are known to occur at the same site.[9,10] Histological examination of a malignant tumour in the scalp showing evidence of keratin production suggests squamous cell carcinoma, a more common tumour at this site.[9] Evidence of trichilemmal keratinization, lobular pattern and the lack of a precursor epidermal lesion such an actinic keratosis differentiates MPTT from squamous cell carcinoma. Since MPTT has a tendency to metastasize and recur more frequently than squamous cell carcinoma, an accurate diagnosis is essential.

Malignant proliferating trichilemmal tumour may further resemble TLC on histology. Trichilemmal carcinoma is a rare malignant skin tumour occurring in older individuals with a predilection to sun-exposed areas. Trichilemmal carcinomas have been reported in solar keratosis, scars of burns, following high-dose radiation exposure and in post-transplant states. Trichilemmal carcinoma often occurs as a papular, nodular or exophytic solitary lesion measuring less than 2 cm in size. Histopathologically, TLC demonstrates a lobular proliferation centred on pilosebaceous structures and is composed of clear cells that are characterized by clear cytoplasm and prominent nucleoli. Trichilemmal carcinoma usually shows continuity with the epidermis or follicular epithelium as a pagetoid interface, exhibits trichilemmal keratinization and dermal invasion.[11–13]

Based on size, clinical details and histology a diagnosis of MPTT was suggested in the present case. Malignant proliferating trichilemmal tumour usually arises as a subepidermal tumour in women over 60 years of age, while the present case report highlights its occurrence in a young lady.[14] Histopathological examinations of MPTT show a variable growth pattern with solid, lobular and trabecular areas. Tumour lobules are sharply circumscribed by a hyaline membrane that is periodic acid Schiff stain (PAS)-positive.[10] Tumour islands consist of large oval to polygonal tumour cells with moderate cytological atypia. Cytoplasm of tumour cells appears clear or eosinophilic with positivity for PAS stain which is diastase sensitive. Focal areas exhibit pilar type of keratinization with peripheral palisading of cells. Some cells show individual cell keratinization and many mitotic figures. Although tumour nests show a pushing border, infiltration into the deep dermis is a notable feature. Necrosis and calcification can also be seen. Magnetic resonance imaging (MRI) of MPTT demonstrates poorly-defined margins with penetration of the tissue planes.[10] Similar to the other adnexal tumours, on immunohistochemistry MPTT expresses cytokeratin 5/6 immunostaining.[15] Normal immunoreactivity pattern for CD 34 noticed in tumours of outer root sheath is lost in MPTT, a feature of undifferentiation.[16]

Treatment modalities differ for TLC and MPTT and a review of the literature reveals the former to be a malignant neoplasm with indolent behaviour, hardly ever resulting in recurrence or metastasis.[12] Surgery is the treatment of choice for TLC with complete excision and periodical follow-up. Wide local excision with a 1-cm margin of normal tissue is the preferred treatment for MPTT. In addition to surgery, chemotherapy and radiotherapy have been used by some authors to prevent recurrence in MPTT.[5] No adjuvant therapy was given to our patient post surgery. However, the patient has been kept under close follow-up and there has been no recurrence one year after the surgery.

### Concluding remarks

Malignant proliferating trichilemmal tumour can rarely occur in the young especially, in an individual with pre-existing proliferating trichilemmal tumour. Since it follows an aggressive course, it is essential to distinguish it from other similar-looking neoplasms for an appropriate therapy.
